# Proteogenomics of selective susceptibility to endotoxin using circulating acute phase biomarkers and bioassay development in sheep: a review

**DOI:** 10.1186/1477-5956-12-12

**Published:** 2014-03-01

**Authors:** Saul Chemonges, John-Paul Tung, John F Fraser

**Affiliations:** 1 The Institute of Health and Biomedical Innovation (IHBI), Queensland University of Technology, Brisbane, QLD, Australia; 2 The University of Queensland, Brisbane, QLD, Australia; 3 Research and Development, Australian Red Cross Blood Service, Brisbane, QLD, Australia

**Keywords:** Proteogenomics, Proteomics, Endotoxin, Circulating biomarkers, Acute phase proteins, miRNA, Sheep

## Abstract

Scientists have injected endotoxin into animals to investigate and understand various pathologies and novel therapies for several decades. Recent observations have shown that there is selective susceptibility to *Escherichia coli* lipopolysaccharide (LPS) endotoxin in sheep, despite having similar breed characteristics. The reason behind this difference is unknown, and has prompted studies aiming to explain the variation by proteogenomic characterisation of circulating acute phase biomarkers. It is hypothesised that genetic trait, biochemical, immunological and inflammation marker patterns contribute in defining and predicting mammalian response to LPS. This review discusses the effects of endotoxin and host responses, genetic basis of innate defences, activation of the acute phase response (APR) following experimental LPS challenge, and the current approaches employed in detecting novel biomarkers including acute phase proteins (APP) and micro-ribonucleic acids (miRNAs) in serum or plasma. miRNAs are novel targets for elucidating molecular mechanisms of disease because of their differential expression during pathological, and in healthy states. Changes in miRNA profiles during a disease challenge may be reflected in plasma. Studies show that gel-based two-dimensional electrophoresis (2-DE) coupled with either matrix-assisted laser desorption/ionisation time-of-flight mass spectrometry (MALDI-TOF MS) or liquid chromatography–mass spectrometry (LC-MS/MS) are currently the most used methods for proteome characterisation. Further evidence suggests that proteomic investigations are preferentially shifting from 2-DE to non-gel based LC-MS/MS coupled with data extraction by sequential window acquisition of all theoretical fragment-ion spectra (SWATH) approaches that are able to identify a wider range of proteins. Enzyme-linked immunosorbent assay (ELISA), quantitative real-time polymerase chain reaction (qRT-PCR), and most recently proteomic methods have been used to quantify low abundance proteins such as cytokines. qRT-PCR and next generation sequencing (NGS) are used for the characterisation of miRNA. Proteogenomic approaches for detecting APP and novel miRNA profiling are essential in understanding the selective resistance to endotoxin in sheep. The results of these methods could help in understanding similar pathology in humans. It might also be helpful in the development of physiological and diagnostic screening assays for determining experimental inclusion and endpoints, and in clinical trials in future.

## Introduction

During subsequent studies based on an ovine model of transfusion-related acute lung injury [1-3], unpublished observations showed that some sheep were more susceptible to the effects of *Escherichia coli* lipopolysaccharide (LPS) endotoxin than others from the same mob. Endotoxin LPS from *E. coli* serotype O55:B5 was infused into sheep to prime their immune system without causing LPS-induced acute lung injury. As a result, the more susceptible sheep required considerably less LPS to prime their immune system. It is not known why this observation occurred, despite the sheep being the same breed, with similar characteristics. This striking observation of differential susceptibility to LPS has prompted studies that seek to explain the variation. It has recently been proposed that tolerance to endotoxin is one mechanism among others involving reprogramming of immune system cells to regulate inflammation and protect the host against shock [4]. Sheep play a vital role when used as large animal models in understanding human disease [5]. To date, however, data on the use of sheep is scarce and yet these production animals fulfil the criteria in the quest for clinically relevant large animal models that can generate robust mechanistic information that lends itself to translational research. Current evidence from landmark genomic studies points out that mouse models that have traditionally been used in research are remote from human conditions [6], which underscores the relevance of sheep. Recent advances in the mapping of the sheep genome and breed variation [7] may help to further elucidate genetic and phenotypic differences seen during natural and experimental disease challenges. We have formulated the hypothesis that the genetic trait, biochemical, immunological and inflammation marker patterns of the host contribute in defining and predicting the response to the bacterial endotoxin (LPS) challenge.

The focus of the current review is on the proteogenomic approaches of studying circulating acute phase biomarkers that could help to explain selective susceptibility to endotoxin in sheep. On the same theme, the effects of endotoxin in animals and understanding of immunity and infection are outlined. The genetic basis of innate defences, the activation of the acute phase response (APR), and the different methodologies employed in detecting novel biomarkers specifically acute phase proteins (APP) and microRNAs (miRNAs) in serum or plasma are discussed. Acute phase proteins are noted as excellent candidates to study in animal models of disease [8]. The inclusion of circulating miRNAs is a novel approach as they have been proposed to play a key role in the modulation of endotoxin tolerance as reviewed recently [4]. The methodologies discussed include routine haematology and biochemistry panels, contemporary proteomic and high throughput genomic approaches. With *omics* (branch of study in life sciences with the suffix -omics, such as genomics, proteomics [9,10]) methods, it can be advanced that it will be feasible to understand the molecular mechanisms and gene expression traits responsible for the selective susceptibility to endotoxin and disease challenge in sheep. An understanding of associated markers of the selective morbidity of sheep to endotoxin may help to understand or predict similar pathology in humans. Furthermore, this knowledge could be used in developing strategic bioassays for use in physiological screening of animals prior to recruitment in experimental studies, and, most importantly, for improving animal welfare and production.

### A - Endotoxin: the molecular trigger of the immune system

An endotoxin is a bacterial toxin that is integral and major part of the cell wall of Gram-negative (G-ve) bacteria. It is made of lipopolysaccharides (LPS), which are biologically-active molecules that bind either directly to the plasma membrane of mammalian cells, or complex with LPS-binding protein (LBP) prior to endocytosis [11,12]. LPS are localised in the outer layer of the membrane and in non-capsulated bacteria, it is exposed on the cell surface [12]. In G-ve bacteria, the lipopolysaccharide membrane protects the bacterium against the action of lipophilic antibiotics [12] and detergents. Bacterial endotoxins are considered to cause the most pathophysiological reactions during infection [13]. LPS endotoxins are key factors in septic shock in humans [14,15], induce strong immune responses in normal mammalian cells [12,16] and are known to be an important cause of acute respiratory distress syndrome in both humans and animals [17]. The lipid A component of LPS is primarily responsible for immune response activation [18]. When G-ve bacteria cells are lysed by the immune system, particles of the membrane containing lipid A are released into the systemic circulation, leading to fever, sometimes diarrhoea, and endotoxic shock or sepsis with potentially fatal consequences [14,15,18,19].

How do mammalian hosts respond to endotoxin? LPS constitute microbial structures called pathogen associated molecular patterns (PAMPs) that are recognised by blood- and cell-associated protein triggering inflammation [20] through a precise sequence [21]. The innate inflammatory response to LPS is mediated via toll-like receptors 4 (TLR4) [4,16,22]. In circulation, LPS is identified and bound by LBP which delivers LPS to the membrane-bound receptor called cluster of differentiation 14 (CD14), creating CD14/TLR4/LBP complex [19,23]. When engaged by LPS, as in Gram-negative infection, this complex (CD14/TLR4/LBP) transduces a signal detected by the myeloid differentiation primary response gene (MyD88) and is passed onward by a cascade of the interleukin-1 receptor-associated kinases (IRAKs), tumour necrosis factor receptor associated factor 6 (TRAF6), and nuclear factor (NF)-kappaB inducing kinase (NIK), resulting in activation of NF-kappaB [4,23]. This promotes the secretion of pro-inflammatory cytokines including interleukin 6 (IL-6), tumour necrosis factor-α (TNF- α) and interleukin-1β (IL-1β) [12,21,24]. Cytokines are target genes of LPS signalling and are activated or downregulated via these transcription factors [16]. The production of pro-inflammatory cytokines and chemokines then finely regulates the systemic reaction events [21]. Readers are referred elsewhere [25-27] for detailed schematic illustrations of TLR4 pathway signalling.

Lipopolysaccharide preparations continue to be the mainstay of experimental models of infection, but why only in small animals? The good news is that studies are starting to emerge that have used large animals in research for the elucidation of LPS immunology [28], blood transfusion studies [2,3], physiology and many other processes to induce synthesis and secretion of intermediary factors such as interleukins [12,29], and in the study of acute phase response [13] in animals. In the quest to characterise the acute phase response, Kabaroff et al. [19] challenged ewes with a range of doses of *E. coli* endotoxin. Their study observed that the expression of TLR4, CD14, IL-6, TNF- α, IL-1β, macrophage migration inhibitory factor, 11-β-hydroxysteroid dehydrogenase and tachynin precursor 1 hepatic genes was dependent on both the dose and the kinetics of the response to LPS [19]. It would have been interesting to determine if there were any soluble gene expression transcripts in plasma and serum. In mice, for instance, it has been shown that the key factors responsible for the sensitivity to endotoxin infusion are found in serum, not the host cells [30]. This information is lacking in sheep, therefore warranting investigation. The identification and harnessing of these circulating factors may help to develop strategies that can dampen response to bacterial diseases [31] in sheep. Whilst there is still debate as to whether animal models of endotoxaemia are relevant to human sepsis, as reviewed by Seok et al. [6], Ward [20] and Munford [31], the use of LPS to simulate infection by priming the immune system of the host and to measure immunological and markers of inflammation in serum of sheep will undoubtedly open doors to this unexplored area of data-scarce large animal species. It has been documented that animals that survive an exposure to endotoxin or Gram-negative bacteria often develop tolerance to subsequent challenges with LPS [31]. This may, in part, help to explain why there is differential resilience to the effects of endotoxin in addition to immunity and genetics of the individual – which is the premise for this review.

### B - Infection, immunity and the role of genetics

The fundamental definition of disease resistance includes freedom from clinical signs of disease after challenge [32]. Animals resist infection by way of innate and adaptive immunity strategies. Innate immunity involves physical barriers like the skin, innate immune cells such as dendritic cells, monocytes, natural killer cells, lymphocytes and inflammatory cytokines [8,33]. The injection of an endotoxin intravenously may therefore not simulate what happens in natural infections as this avoids the acute phase of infection [31] and traverses some of the innate immunity barriers. It is well recognised that a heightened tolerance to pathogens is an important selection objective in most livestock species. Also, animals that survive an exposure to endotoxin or Gram-negative bacterial infection often develop tolerance to subsequent challenges with LPS and other microbial agonists [31]. This is by way of adaptive immunity which is antigen-specific and requires the recognition of specific antigens via a process of antigen presentation that results in an immunological memory [31,33].

Studies have also shown that various innate and adaptive immune traits are genetically controlled, and resistance to disease is an evolutionary trait responsible for survival [33]. Most work on sheep has shown that variation in resistance to internal parasites, fleece rot, fly strike, and dermatophilosis is genetically determined [32]. In animal production, the relative importance of infectious diseases depends on their impact on welfare and production; genetic approaches to disease control have only been considered where conventional control measures have been ineffective [32], which leaves considerable knowledge gaps in this field. Along this line, Albers et al. [34], have been able to recognise and estimate genetic relationships among resistance and resilience to internal parasite burdens as being important production characters in sheep. Their study deduced that realistic additions to existing selection criteria for Merino sheep should include productivity when infected, and resistance to infestation [34]. Furthermore, the potentially negative impact of reduced selection efficiency on production traits has also been observed in sheep in some reports [32]. This could be elucidated by understanding the mechanisms underpinning the resistance, the intricate genes and the use of biomarker-assisted selection [32]. In other species like wild rodents, for example, phenotypic variation in immune function and pathogen resistance has been shown to occur in natural populations [35,36], which strengthens the notion that there is a genetic basis to this variation. In summary, infection, the immune system and the genetic factors all interact to mount an acute phase response (APR).

### C - Acute phase response: the humoral gatekeeping signaling

The physiological response to infection and injury involves inflammation, in the first instance, leading to the activation of the body’s immune system-mediated defence mechanisms, collectively referred to as APR [21,37-39]. The APR is an innate, non-specific reaction which occurs in animals shortly after an infection, inflammation, trauma, surgery neoplasia or immunological disorders prior to specific immunity development [39,40]. Innate immunity therefore fulfils an important role in the body’s early defence by initiating APR that triggers the production of blood acute phase proteins (APP) mainly by hepatocytes [8]. The production of APP is regulated by specific genes [41]. Other than hepatocytes, several other tissues and cell types are known to make APP as well [21]. APR is a multifaceted systemic primary defence system activated by trauma, infection, stress, neoplastic processes and inflammation [8,21]. The APR is a core part of the innate immune response and is observed across all animal species [8,38]. The APR is mediated mainly by pro-inflammatory cytokines [40], acting as messengers between the local site of injury and the cells that synthesise APP [38]. As far as disease resistance in sheep is concerned, Raadsama et al. [32] asserted that genetic variations exist in acute phase responses in almost every case that has been investigated. Therefore, continuous focus on alternative selection criteria including biomarkers such as DNA [32], APP and miRNA are warranted. The latter two novel additions and products of the APR have a tremendous potential as biomarkers to enhance the understanding of host-pathogen relationships and provide new avenues for developing tools for mitigating the effects of disease.

### D - Biomarkers: the pathognomonic yardsticks for biological processes

A biomarker is an objective, quantifiable characteristic of a biological process [42]. Biomarkers include established measurable physiological parameters like body temperature, heart rate, respiratory rate (the TPR adage), haematological & biochemical parameters, acute phase proteins, and gene expression transcripts like cytokines and novel miRNAs all of which are necessary for investigations of the systemic impact of disease. The effects of disease cause physiological derangements that can be measured by haemodynamic, respiratory and temperature monitoring technology. Detailed insights on the practices of haemodynamic monitoring practices are beyond the scope of this review and can be found elsewhere [43-48]. Respiratory monitoring is not only about checking if the chest is moving; it is more about the analysis of actual pulmonary gas exchange. Gas exchange can be quantified by pulse oximetry which provides a measure of oxyhaemoglobin saturation in blood and is one of the most useful parameters [49-52] in addition to the measurement of end tidal carbon dioxide tension. Blood gas analysis provides information on partial pressures of O_2_ and carbon dioxide. Absolute values for oximetry including the concentrations of total blood haemoglobin, oxygen saturation, fractions of oxyhaemoglobin, methaemoglobin, carboxyhaemoglobin, and de-oxyhaemoglobin are also attainable by blood gas analysis. Modern blood analysers also provide measurements for blood acid-base status, electrolytes and metabolites. Complete blood cell counts including platelets, differential white cell counts and serum or plasma biochemical analysis [53] continue to be the mainstay of routine biomarker benchmarks of organ function in practice.

## Acute phase proteins

APP are large structurally unrelated proteins [21] that are considered biomarkers, and their assay can provide an evaluation of the activating event [8,54-56]. It is also known that APP are sensitive markers of infection and inflammation in animals [8,38]. Later in APR, innate responses are reliant on cytokines and chemokines which are generated by activated cells including fibroblasts, macrophages, platelets, monocytes, endothelium, keratinocytes and T-cells [8].

The fundamental objective of studying APP is to understand the pathophysiology of disease processes involved during innate immune response to infections in order to improve the welfare of animals [21]. Their characterisation has potential in medical interventions and in monitoring disease processes. It has been proposed that, as in humans, the monitoring of systemic inflammation in veterinary patients could be achieved by the determination of APP profiles [57]. The latter may in fact be useful in monitoring the health status of animals during farm production, slaughter [40,58] and agistment in the case of experimental animals, as well as monitoring their health status. Cross-species comparisons have indicated a considerably high degree of similarity in the patterns of APR [39]; however, although APR is highly conserved in nature, APP profiles show a significant variability between species [59]. In other words the relative level or magnitude of expression of different APP varies depending on the species. It has been established that APR leads to fever, leucocytosis, significant alterations of APP in plasma or serum [21,38] and increased numbers of circulating neutrophils and their precursors [38].

More than 200 APP have been recognised and are grouped as either positive or negative, contingent on whether they increase or decrease, respectively during APR [8,21,38]. Albumin is the major negative APP that decreases in nearly all animal species during APR [8,21,38]. The downregulation of hepatic synthesis of albumin increases the free amino acid pool so they can be available for gluconeogenesis [21]. Positive APP are those that increase during APR and can be further categorised as major, moderate or minor depending on the degree of increase during APR [8]. There are species differences in the classification of positive APP [38]. In sheep, for example, the major APP (>10-fold increase) are haptoglobin and serum amyloid A, while α1-acid glycoprotein (AGP) and C-reactive protein are moderate APP (1- to 10-fold increase) [8]. In humans, C-reactive protein (CRP), the APP of main interest, and serum amyloid A are major APP, while AGP, fibrinogen and haptoglobin are moderate APP [8]. Serum amyloid A is consistently a major APP in the cat, ox, dog, goat, horse, man, mouse, pig, and rabbit, but not in the chicken, non-human primates and rat [8]. As an example from another species, Orro et al. [13] demonstrated that challenge with *E. coli* endotoxin in reindeer can activate APR, and serum amyloid A appears to be a more sensitive indicator of APR than haptoglobin in this species [13]. It is known that different pathophysiological challenges generate different patterns of APP; therefore, not all of them change uniformly in all diseases or in each animal [21]. A wider application of APP in veterinary medicine has only heightened in the last decade [8] and a lot remains to be learned. The biologic functions of some common APP are presented in Table 1.

**Table 1:** Biologic functions of some common acute phase proteins (APP)

**Acute phase protein**	**Function**
C-Reactive protein (CRP)	Opsonises infectious agents to activate complement and phagocytosis. Up- or downregulates cytokine production and chemotaxis. Has both pro-inflammatory and anti-inflammatory effects.
Serum amyloid P	Analogue of CRP in some animals.
Serum amyloid A (SAA)	Chemotaxis of polymorphonuclear cells, monocytes, and T cells. Downregulates the inflammatory process. Binds cholesterol and modulates innate immune reactions. Known as an innate immunity opsonin.
Albumin	Binds fatty acid, bilirubin. Regulates osmotic pressure.
Haptoglobin (Hp)	Dampens oxidative damage due to haemolysis by binding free haemoglobin. Bacteriostatic. Immunomodulator. Angiogenesis and chaperone activity.
α_1_-Acid glycoprotein (AGP)	Binds and inhibits LPS. Downregulates neutrophils and complement. Transports molecules in plasma. Immunomodulator of white blood cells. Reduces apoptosis of bovine monocytes. Antibacterial.
α_1_-macroglobulin	Protease inhibitor; removes enzymes released during injury.
Lipopolysaccharide binding protein (LBP)	Binds LPS, activates innate immunity. Modulates biological activity of immune cells. Opsonin.
Inter α trypsin inhibitor H4 (ITIH4)	Protease inhibitor.
Fetuin (α HS glycoprotein)	Bone growth, foetal development
α_1_ Anti proteinase (Anti trypsin)	Protease inhibitor
Major acute phase protein (Porcine) (pig-MAP)	Trypsin inhibitor (porcine species).
Ceruloplasmin	Scavenges free radicals.
Paraoxanase	Oxidase inhibitor.
Lipoprotein	Lipid transport.
Retinal binding protein	Transport of vitamin A.
Mammary-associated serum amyloid A3	Milk APP.
Fibrinogen	Precursor for fibrin, tissue repair.
Transferrin	Positive APP in avian species and negative in mammals. Immunomodulator, protein transport, tissue protection from damage from inflammation.

Table reflects information from references [8,21,37,38].

An extensive summary of the triggering event and the significant changes in APP in animals has been reviewed by Cray et al. [8]. Their review noted that the uses of APP assays are well supported in the human and veterinary studies with many potential uses in laboratory animal medicine [8]. Specific reports on APP alterations in sheep have been published, for instance during bacterial infections [60-62], yeast infection [63] and in an experimental model of bacterial lymphadenitis [64]. In one study, it was noted that ceruloplasmin, fibrinogen and haptoglobin increased and albumin decreased during a yeast infection [63]. Sheep with rhino-tracheo-bronchitis show significant decreases in transthyretin, apolipoprotein A1, and increases in haptoglobin, endopin 1b and alpha 1β- glycoprotein [62]. In cattle, serum haptoglobin concentration is useful in the effective diagnosis and prognosis of enteritis, peritonitis, pneumonia and endocarditis [21]. Research shows that sheep and goats have similar APP responses as cattle [21], but this could still be an assumption. In sheep, AGP shows a moderate, but extended, response after an inflammatory stimulus, suggesting that it is a marker for chronic conditions in this species [64] and goats [65]. Fibrinogen is a moderate APP in small ruminants [65]. It is important that these findings are replicated in order to formulate a standard.

The APP have been reported to be more sensitive than complete blood count analysis as makers of infection particularly in sheep [60]. For example, APP assay can differentiate between acute and chronic states of infectious conditions such as caseous lymphadenitis [64]. In Merino lambs, APP assays have been shown to be useful in evaluating the systemic effects of different procedures for controlling breech flystrike [53,66,67]. This can potentially help to identify the procedures associated with downregulated inflammation [21]. The APP profiling can be used as a prognostic indicator of dystocia in sheep because of elevations in haptoglobin [21]. In comparison to cattle, there is considerably less reported on APP in small ruminants [21], suggesting avenues for robust research focused in this area. Characterisation of APP response during infectious disease in sheep and goats has been identified as an area that would have a significant knowledge impact [21]. In dogs, it has been suggested that ceruloplasmin and fibrinogen levels could have potential in the diagnosis of pregnancy and health status monitoring of pregnant bitches [68]. The current school of thought suggests that a single APP should not be used to monitor a disease process [8,38]. Instead, a comprehensive index [69] that encompasses both positive and negative APP as well as those APP that increase both rapidly and slowly should be employed in order to correlate better with the inflammatory process [8,62]. This calls for the use of contemporary methodologies to develop multiple and comprehensive and rapid APP profiling platforms.

### Cytokines and chemokines

The physiological effects of LPS are based predominantly on the production and activation of molecular mediators such as cytokines [70]. Currently, cytokines are considered to be the principal mediators coordinating physiological response to stress stimuli in health or disease and the immune system [71,72]. They are known for the recognition, modulation and ultimate response of an organism to infection by regulation of activation, replication, chemotaxis and apoptosis of immune cells [73]. The APR is stimulated by the release of cytokines such as IL-1, IL6 and TNF-α from macrophages and monocytes at the site of inflammation or infection [38,70]. Compared to other species such as humans and mice, it is still early days for cytokine research in sheep which has fortunately been enhanced by *omics* techniques [71].

During infection or LPS stimulation, cytokine genes are turned on and a ‘storm’ of cytokines is produced. Under usual circumstances, this storm subsides as the infection is eliminated and the genes are turned off [8,73]. However, when cytokines genes remain persistently activated, this can lead to an overactive immune system and the development of autoimmunity, causing cell death [73]. Cytokines, therefore, dictate the nature of the immune response to infection [71]. It should be acknowledged that intravenous injection of LPS does not mimic any natural Gram-negative infection and can be variable. In mice for instance, the initial response to LPS is pro-inflammatory as TNF-α and IL-1 appear in the blood long before IL-10 does – and yet there is not much evidence to suggest that this happens in naturally infected humans [31]. It is, therefore, important to characterise LPS-induced cytokine expression and profiles in sheep and document any differences.

Cytokine responses are integral to the survival of the host, while in some instances, they also seem to drive the disease process [8]. This dysregulation of the cytokine response is present in a number of disease states, including those that are immune-mediated rather than infectious [73]. Over-exuberant production of pro-inflammatory cytokines causes death of cells, dysfunction of organs and death of the organism [20]. This is known as the systemic inflammatory response syndrome (SIRS) [20].

Each cytokine binds its own receptor and that receptor has a defined mechanism to its second messenger system. Cytokines are not stored. They are expressed as needed and their expression is tightly controlled. It has been documented that cytokines peak at different times during the onset of illness [73]. This makes the use of cytokines as markers for disease severity difficult [73]. Peaking too long or not peaking at all, or in the wrong proportions may be responsible for disease states. The ultimate end product of cytokine production is signal transduction [20]. Table 2 summarises some known cytokines categorised in families and their action.

**Table 2:** Families of some known cytokines

**Family**	**Members**	**Action**
TNF	20	Pro-inflammatory
IL-1	11 Members including IL-1α, IL-1β, IL-18 and IL-33	Pro-inflammatory
IL-6	IL6, Leukaemia inhibitory factor, IL-11, oncostatin, ciliary neuroptic factor and cardiotropin-1	Acute phase response, Pro-inflammatory, diverse biological activities
IL-10	IL-10, IL-22	Anti-inflammatory
Colony stimulating factors (CSF)	IL-3, granulocyte-CSF, granulocyte-macrophage CSF, macrophage CSF	Overlapping functions, distinct gene products, and have specific receptors.
Chemokine	CXCL8 (formally known as IL-8)	Chemotactic
Interferons	IL-2, IL-4, IL-7, IL-9, IL-15, and IL-21	Antiviral, modulate several immune responses, share a common receptor chain, exclusively produced by activated T Cells.

Table reflects information drawn from references [38,71,73].

### Assaying of acute phase proteins

Automated chemistry analysers are commonly used for basic health assessments for total protein and albumin [8]. Currently, however, protein electrophoresis (PEP) is considered as an excellent method for detection for APP [8]. PEP can provide a more accurate albumin quantitation and visualisation of globulin fractions to monitor the overall progression of the APR [8]. The downside of PEP is that it does not enable quantitation of individual proteins, but rather clusters of proteins that are mediators of the acute inflammatory process [8]. It is recommended that baseline parameters for species and strains should be established by each experimental laboratory when using PEP or APP tools [8]. Turbidimetric methods [62], radioimmunoassay (RIA) and ELISA in cattle, for instance [13], continue to be developed specifically for rapid determination of CRP [38]. There are some ELISA kits [68] that have been validated for animal use and are available to measure specific APP, however most of these assays lack automation and could prove to be uneconomical [8]. The development of protein microarray methodology has been suggested for assaying of APP in pigs [38]. Considered together, current evidence suggests that the proteomic approach of APP assay is preferred.

There has been relatively limited use of proteomics to investigate blood protein patterns in relation to APP assays in farm animals [21,62,74]. Proteomic studies in veterinary medicine, particularly of sheep, remain constrained, compared to other species like humans [62]. Proteomic approaches enable large-scale protein analysis, describing the entire protein component (proteome) from an organism, tissue, cell or biological fluid. This then provides information on protein expression, localisation, functions and interactions [62]. There are a few studies that have described two-dimensional electrophoresis (2-DE) in cattle [40,75-77], horses [78], sheep [62], swine [79], chicken [80] and companion small animals [81]. One experimental model used 2-DE in cattle, followed by Liquid chromatography–mass spectrometry (LC-MS/MS) for spot identification to determine potential biomarkers of disease outcomes to predict the occurrence of fatal respiratory disease [82]. Alonso-Fauste et al. [40] used 2-DE coupled with Matrix-Assisted Laser desorption/Ionisation Time-of-Flight Mass Spectrometry (MALDI-TOF MS) for proteomic characterisation of bovine serum and whey from healthy and mastitis-affected cattle. They concluded that existing differences in serum protein patterns between healthy and acute phase conditions is very useful in the search for specific biomarkers. Moreover, recent studies by Chiaradia et al. [62] defined the sheep proteome and identified protein markers that recognise early, and subclinical disease. Their study concluded that applying proteomics in putative biomarker discovery for early diagnosis, as well as monitoring the physiological and metabolic situations, is critical for ovine welfare [62]. The downside of investigating the plasma proteome, however, is that few proteins of high abundance can mask changes in proteins of lower abundance [83]. While it has been suggested that it is necessary to deplete major proteins, including high abundance APP from samples prior to undertaking proteomic investigations of low abundance proteins [21], other studies [62] have objected to sample pre-treatment such as dealbumination. Developments in this field suggest that proteomic investigations are gradually shifting from gel-based 2-DE to non-gel based studies where LC-MS/MS approaches are able to identify a wider range of proteins which may overcome the problem of high abundance proteins [21]. Recently, an approach for proteome analysis has been developed that combines LC-MS/MS and data extraction strategy called sequential window acquisition of all theoretical fragment-ion spectra (SWATH) or SWATH-MS [84]. SWATH-MS has been suggested for use in plasma biomarker discovery studies [85].

The profiles of cytokines can be measured at two levels, protein or mRNA transcripts using various techniques [72]. In a study on brachycephalic dogs that assessed concentrations of pro-inflammatory and anti-inflammatory cytokines in plasma, a specific commercial ELISA kit was used satisfactorily [86]. In another study, ELISA was used to measure TNF and IFNγ levels in cell supernatants following LPS stimulation in bovine peripheral blood mononuclear cells (PBMC) [16]. The latter study concluded that LPS stimulation alters the expression of genes encoding epigenetic enzyme in bovine PBMC [16]. Real-time quantitative RT-PCR is the method commonly used for cytokine gene expression in tissues and has been used in sheep [72,87-89]. The qRT-PCR allows for an exponential amplification of the gene of interest; therefore, overcoming the sensitivity problems usually encountered with gene expression studies [72]. Owing to the lack of immunological and biological assays for the detection of ovine cytokines, Budhia et al. [72] developed the use of qRT-PCR to detect ovine cytokines. Micro-array methodologies for cytokine assaying for ruminants have now also been developed [90].

Many potentially novel applications of APP have been proposed. APP assays could be used as rapid screening tests for inflammation by providing better sensitivity when compared to other traditional markers [91]. By replacing the currently used subjective assessments, APP may be useful in defining human endpoints to experimental protocols and also to gauge stress with regard to animal wellbeing [8]. Studies on APP have shown that optimum husbandry conditions and isolation of laboratory animals minimises contact with potential triggers of inflammation [54]. Therefore, another potential use of APP is the assessment of the general health of laboratory animal colonies and to screen animals before entry into experimental protocols [8]. Due to the observations of the dynamic APP response in sheep following vaccination, it has been suggested that exploitation of this effect would provide a means to monitor the APP and innate immune response in the general population without the need for primers such as LPS [21]. Cray et al. [8] summed it up elegantly that APP are excellent candidates through which to better study animal models of disease, monitor health and objectively assess animal wellbeing. There is undoubtedly a considerable potential for applying proteomic studies to ovine plasma or serum to further understand APR in the pathophysiology of disease, to benefit the health and welfare of sheep, be it in farm production or as large animal models of human disease.

## Circulating microRNA: the future of biomarkers

The development of functional genomics has created a considerable interest in discovering circulating biomarkers that are useful in classifying disease severity and in gauging therapeutic response to candidate drug interventions [92]. miRNAs are newly discovered, small non-coding ribonucleic acids (RNAs) about 22 nucleotides in length that play critical roles in the regulation of host genome expression at the post-transcriptional level [93-95] and are thought to impart stability to biological systems [96]. Early observations on the *lin-4* gene that codes for short RNAs governing the development of *Caenorhabditis elegans* were first reported by Lee et al. [97] in 1993. In later years, another short RNA *let-7* was identified [98], with a large number of small RNAs being revealed in 2001 [92], which subsequently opened the lid to the world of the microRNA biotechnology [93,99-102]. Recently, Zhang et al. [103] identified and characterised the miRNA transcriptome of sheep. The identification of miRNA will promote the study of miRNA functions and gene regulatory mechanisms [103] and networks will be subject of many advances in miRNA biology in the coming years [92].

miRNAs are detectable in a variety of sources, including tissues, serum, plasma and other body fluids [104] and have been reported to be remarkably stable even under conditions as harsh as boiling, extreme pH, long-time storage at room temperature and multiple freeze-thaw cycles [105,106], therefore prompting interest in this line of research [104,107]. Much as the origin of cell-free miRNAs in circulation has not been clearly elucidated; they are believed to be released to the extracellular space from damaged or apoptotic cells and are also actively secreted by cells via exosomes or exocytosis [108-110]. They are protected against degradation by being within lipid vesicles or by being associated with protein or lipoprotein complexes [107]. Furthermore, miRNA expression is known to be specific in different tissues [111] and change in different diseases [112,113]. Thus, miRNAs in plasma or serum could be developed as a novel class of blood-based biomarkers to diagnose and monitor disease [107,109,114].

The work of Montano [92] observed that there is a growing recognition that miRNA networks are often associated with tissue dysfunction and are likely to be a key source of altered gene expression that distinguishes healthy tissue from pathological tissue. For example, in diabetic humans, it has been shown that there is an apparent discordance between proteomic profiles and their respective miRNA, suggesting a potential role played by miRNAs [115]. Quite recently, there have been detailed insights into the function of miRNAs in various disease states such as liver disease [116], kidney function and disease [117], cardiac disease [118], therapeutic targets in cancer [100], biomarkers in lung cancer[119], lung cancer biology and therapy [120], pulmonary fibrosis [121], acute lung injury [122] and in understanding immunoregulation, inflammation and autoimmune diseases [93].

Based on the preceding background, it is reasonable to assume that changes in miRNA profiles occur in sheep during LPS challenge which may be reflected in plasma. It is now known that miRNAs are post-transcriptional regulators of gene expression and are new targets for revealing the molecular mechanism that form traits [103]. There has been a pioneering study as discussed [93,122] that links miRNAs with innate response based on the observation that miR-146 and miR-155 are rapidly up-regulated during LPS stimulation of human monocytic cells [92]. In addition, there is also mounting evidence that miRNA have a critical role in immune system homeostasis [93]. Studies are therefore indicated to characterise the acute phase response by measuring miRNA expression as potential biomarker candidates in sheep challenged with LPS. This could in part, help to explain the differential resilience to endotoxin that has been observed in sheep. It has been proposed that further study of miRNAs will prove beneficial in the understanding of physiological and pathological mechanisms in organisms, thus providing a theoretical basis for the diagnosis and treatment of diseases [103].

### Assaying of miRNAs

The detection and quantification of miRNAs in plasma and serum samples is an area that continues to grow [109,123-125]. In brief, the methods commonly used quantify miRNAs are quantitative reverse-transcriptase polymerase chain reaction (qRT-PCR) profiling and micro-array technology [107]. One study [123] revealed that vesicle-associated plasma miRNA was in the minority, whereas potentially, up to 90% of miRNAs in circulation are present in the non-membrane bound form. The relative ease by which miRNAs can be detected in a qRT-PCR and microarrays makes it immensely motivating to study circulating miRNAs as clinical biomarkers in a sheep model following an LPS challenge. Circulating miRNAs are known to fulfil a number of criteria that an ideal biomarker has as discussed and summarised by Creemers et al. [107]. Most importantly, circulating miRNAs can be accessed by non-invasive methods. Furthermore, evidence continues to mount that while circulating miRNAs are quite tissue- or pathology-specific for given tissue pathologies, and their sequences are evolutionarily conserved [107], they may also have the potential to provide the much-needed intermediate endpoints for animal experimental studies. It would also be interesting to determine whether candidate miRNA profiles in sheep correlate with some of the published levels of plasma proteins and cytokines.

There are challenges associated with the detection of circulating miRNAs. There are low numbers of total RNA molecules in blood fluid compartment, which raises concerns with measuring the concentration and quality of isolated RNA [107]. It has been proposed that it is important to precisely normalise [125] detected miRNA values for variances based on the amount of starting material and miRNA extraction, by seeking a “housekeeping” circulating RNA, for instance [107]. Plasma or serum volume has been proposed as the best parameter by which to standardise the amount of input miRNA [126]. Similarly, the number of molecules per volume of plasma or serum is also used as a standard to evaluate blood levels of other molecules [107]. In addition to normalisation of qRT-PCR data, an appropriate control is a requirement for diagnostic studies [125]. Together, these observations mean that studies are indicated to characterise the different normalisation methods and find the best ways to reproducibly measure miRNA in plasma [107] especially in data-scarce and equally important large animal species such as sheep.

Next-generation sequencing (NGS) technologies are regarded as the next step to be used for discovering novel miRNAs as they are not biased by qRT-PCR and microarray platforms [127]. Combining multiple miRNAs into a miRNA profile may provide greater accuracy than a single miRNA [107]. With only approximately 100 miRNAs characterised in human plasma thus far, profiling all the miRNA may provide a comprehensive analysis of pathologies in multiple organs, even in sheep. With respect to sheep, no real-time or NGS has been performed to characterise circulating miRNA expression in response to LPS challenge, therefore calling for research in this area.

### E - Perspectives

Genomic tools and approaches in sheep have lagged behind and have been dwarfed by more influential, and yet equally significant farm animals such as cattle if looked at from a functional genetics point of view [128]. Heightened interest in the understanding of sheep has led to new evidence that supports their relevance and suitability as experimental models for understanding critical illnesses in humans as recently reviewed in related studies [129]. Improved production traits in meat, milk and fine wool currently constitute the predominant spectrum of the phenotypes of modern breeds of sheep at the expense of original strains [7]. It is also now known that conventional genetic improvement programmes aimed at increasing profitability have found it difficult to incorporate disease resistance in multi-trait breeding objectives [128]. Previous studies have largely been on European-derived breeds of sheep which prompted the recent landmark study on diverse breeds across the world to better understand the genetic composition and history of sheep [7]. The study genotyped the global diversity of sheep using the ovine SNP50 Bead chip technology and deduced that, among other traits, the most robust selection signal occurred in response to breeding for the absence of horns [7]. Could the focus on selective breeding for polled sheep have led to the propagation of more disease-prone sheep?

## Conclusions

The current review has established a framework for understanding selective susceptibility to infection with reference to endotoxin through a proteogenomic approach. The identification of unique signature and disease-related APP and miRNAs provides the foundation for the next phase of studies: 1) to mechanistically understand the pathogenic contribution of endotoxin-related APP and miRNAs, 2) to determine the cause and mechanism underlying APP and miRNA dysregulation in experimental LPS challenge and stress for example during transportation, and 3) to ultimately develop novel APP and miRNA-based biomarker assay with potentially unrivalled specificity and sensitivity. To address these central questions, a relevant large animal model of infection is instrumental. It is anticipated that, in future, to treat infectious or stress-related diseases, novel effective APP and miRNA-based gene therapies will be developed to replace the traditional currently available therapies, which are sometimes unsatisfactory or have undesirable side effects.

## Ethical approval

Animal ethics approval for the sheep studies referred to in the manuscript was obtained from the University Animal Ethics Committee of the Queensland University of Technology (QUT) – reference 0800000555 and ratified by The University of Queensland.

## Abbreviations

miRNAs: Micro ribonucleic acids; LPS: Lipopolysaccharide; APR: Acute phase response; 2-DE: Gel-based two dimensional electrophoresis; MALDI-TOF: MS matrix-assisted laser desorption/ionisation time-of-flight mass spectrometry; LC-MS/MS: liquid chromatography-mass spectrometry; SWATH: Sequential window acquisition of all theoretical fragment-ion spectra; SWATH-MS: Sequential window acquisition of all theoretical fragment-ion spectra-mass spectrometry; ELISA: Enzyme-linked immunosorbent assay; qRT-PCR: Quantitative real-time polymerase chain reaction; PAMPS: Pathogen associated molecular patterns; TLR4: Toll-like receptors 4; LBP: Lipopolysaccharide binding protein; MyD88: Myeloid differentiation primary response gene 88; IRAKs: Interleukin-1 receptor-associated kinases; TRAF6: Tumour necrosis receptor-associated factor 6; NF: Nuclear factor; NIK: Nuclear factor-kappaB inducing kinase; TNF,- α: Tumour necrosis factor alpha; APP: Acute phase protein; CRP: C-Reactive protein; AGP: Acid glycoprotein; DNA: Deoxyribonucleic acid; TPR: Temperature pulse-rate, respiration; PEP: Protein electrophoresis; RIA: Radioimmunoassay; IL: Interleukin; SIRS: Systemic inflammatory response syndrome; RNA: Ribonucleic acid; NGS: Next generation sequencing.

## Competing interests

The authors declare to have no competing interests.

## Authors’ contributions

CS, TJ-P and FFJ contributed in the conception and approval of this manuscript. CS and TJ-P performed the experiments for the unpublished experimental work on the sheep studies referred to. CS drafted and wrote the manuscript. All authors read and approved the final manuscript.
